# Comparative analysis of hyperfibrinolysis with activated coagulation between amniotic fluid embolism and severe placental abruption

**DOI:** 10.1038/s41598-023-50911-w

**Published:** 2024-01-02

**Authors:** Rui Ide, Tomoaki Oda, Yusuke Todo, Kenta Kawai, Masako Matsumoto, Megumi Narumi, Yukiko Kohmura-Kobayashi, Naomi Furuta-Isomura, Chizuko Yaguchi, Toshiyuki Uchida, Kazunao Suzuki, Naohiro Kanayama, Hiroaki Itoh, Naoaki Tamura

**Affiliations:** Department of Obstetrics & Gynecology, Hamamatsu University School of Medicine, 1-20-1, Handayama, Higashi-ku, Hamamatsu, 431-3192 Japan

**Keywords:** Reproductive signs and symptoms, Reproductive disorders

## Abstract

Amniotic fluid embolism (AFE) and placental abruption (PA) are typical obstetric diseases associated with disseminated intravascular coagulation (DIC). AFE is more likely to be complicated with enhanced fibrinolysis than PA. AFE may have an additional mechanism activating fibrinolytic cascade. We aimed to compare the coagulation/fibrinolysis factors among AFE, PA, and peripartum controls. We assessed AFE cases registered in the Japanese AFE Registry, and PA cases complicated with DIC (severe PA) and peripartum controls recruited at our hospital. The following factors in plasma were compared: prothrombin fragment 1 + 2 (PF1 + 2), plasmin α2-plasmin inhibitor complex (PIC), tissue factor (TF), tissue plasminogen activator (tPA), annexin A2 (AnnA2), total thrombin activatable fibrinolysis inhibitor (TAFI) including its activated form (TAFIa), and plasminogen activator inhibitor-type 1 (PAI-1). PF1 + 2 and PIC were markedly increased in both AFE (n = 27) and severe PA (n = 12) compared to controls (n = 23), without significant difference between those disease groups; however, PIC in AFE showed a tendency to elevate relative to PF1 + 2, compared with severe PA. AFE had significantly increased tPA and decreased total TAFI levels compared with severe PA and controls, which might be associated with further plasmin production in AFE and underlie its specific fibrinolytic activation pathway.

## Introduction

Disseminated intravascular coagulation (DIC) is a clinicopathologic syndrome that can be initiated by underlying diseases or conditions and develops a systemic intravascular activation of blood coagulation leading to hemostatic abnormalities and/or multiorgan failure^1^. Amniotic fluid embolism (AFE) and placental abruption (PA) are typical obstetric diseases associated with DIC. The incidence of these diseases is rare, 5.0 cases in 100,000 deliveries in AFE^2^, and 0.4% of all pregnancies in PA complicated with DIC^3^; nonetheless, these diseases are frequently associated with maternal and/or fetal morbidity and mortality due to maternal cardiopulmonary and placental insufficiency. Fibrinogen levels were reported to decrease below 70 mg/dL in the early stage before considerable external bleeding in fatal cases of AFE^4^ and PA^5^, which indicates that AFE and PA may induce consumption of coagulation factors by systemic activation of blood coagulation leading to hemostatic disorder.

AFE cases have been reported to show whole blood coagulation trace narrowing earlier after achieving the peak of tracing in thromboelastography (TEG)^6^ and rotational thromboelastometry (ROTEM)^7^. Furthermore, in a previous study, we reported that AFE developed DIC coincided with enhanced fibrinolysis due to increased amount of plasmin, which could further aggravate systemic bleeding diathesis with degradation of the fibrin clot essential for hemostasis^8^. On the other hand, case reports^9,10^ and a prospective study^11^ involving PA complicated with coagulopathy did not show the characteristic TEG traces and/or parameters representing the hyperfibrinolytic state, but only tracing induced by depletion of coagulation factors. These studies suggest that AFE may involve a specific feature of DIC with hyperfibrinolysis, so-called “hyperfibrinolytic DIC”^12^, compared to DIC developed in PA. However, the mechanism and why dominant fibrinolytic reaction is usually observed in AFE rather than PA have not been fully elucidated. Therefore, in the present study, we assessed the difference in activation of blood coagulation and fibrinolysis among AFE, PA complicated with DIC, and peripartum subjects as a control. We hypothesized that AFE has a specific blood coagulation and fibrinolysis cascade that induces hyperfibrinolysis, which is not associated with PA. We aimed to compare coagulation and fibrinolysis factors among AFE, PA complicated with DIC, and peripartum controls.

## Results

### Clinical characteristics of the subjects

Among a total of 2059 cases enrolled in the Japanese AFE Registry, we identified 789 AFE patients who met the definition of DIC by Erez’s diagnostic criteria. Of these, plasma samples before blood transfusion were available for 27 cases. Thus, we recruited 27 AFE, 12 severe PA (sPA) patients, and a total of 23 peripartum control participants, which consisted of patients before labor (n = 9), during labor (n = 7), and postpartum (n = 7). Table 1 shows the patients’ backgrounds. The number of cases complicated with preeclampsia was 99 out of 2059 enrolled cases in the Japanese AFE Registry, and we finally found 0 and 1cases affected by preeclampsia in AFE and sPA groups, respectively, after identification of the eligible study population (Supplementary Table S1). Gestational age at delivery in the sPA group was significantly smaller than the subgroups of During labor and Postpartum in the control and AFE groups. All patients in the sPA group developed their symptoms before onset of delivery, whereas those in the AFE group had onset during delivery and postpartum in most cases. Patients in the AFE group frequently developed cardiac arrest as a clinical symptom. The plasma samples were drawn at 60 and 105 min as the medians after the onset in the AFE and sPA group, respectively, with no significant difference between the groups. There was no significant difference in blood loss amounts between the AFE and sPA groups; however, they were significantly larger than the control group. The amounts of fluid administered before the plasma collection were 1300 and 1355 mL as the medians in the AFE and sPA group, respectively, which were significantly larger than the subgroups of Before labor and During labor in the control group. Background hematological data are described in Table 2. We found that AFE and sPA patients had low platelet counts as well as disturbed coagulation function due to the very low values of Clauss fibrinogen with elevated D-dimer levels compared to the control group; however, there was no significant difference between the two patient groups. There was no difference in the number of DIC patients with the AFE and sPA groups using the diagnostic criteria of overt DIC defined by Erez or the criteria defined by Clark.

**Table 1:** Patients’ backgrounds.

Group	Control						AFE (n = 27)		sPA (n = 12)		P value
	Before labor (n = 9)		During labor (n = 7)		Postpartum (n = 7)						
Age (year)	33	[28–45]	28	[18–38]	27	[23–32]	35	[25–43]	34	[21–42]	< 0.01^a^
Primipara (case)	2	(22.2%)	3	(42.9%)	3	(42.9%)	7	(25.9%)	6	(50.0%)	0.52
Preeclampsia (case)	0	(0%)	0	(0%)	0	(0%)	0	(0%)	1	(8.3%)	0.38
Gestational age at delivery (day)	267	[255–281]	277	[261–287]	277	[267–282]	274	[179–292]	242	[191–271]	< 0.01^b^
Mode of delivery (case)											
Vaginal	2	(22.2%)	7	(100%)	6	(85.7%)	15	(55.6%)	2	(16.7%)	< 0.001^c^
Cesarean	7	(77.8%)	0	(0%)	1	(14.3%)	12	(44.4%)	10	(83.3%)	
Onset (case)											
Before labor	NA		NA		NA		3	(11.1%)	12	(100%)	< 0.01^d^
During labor	NA		NA		NA		8	(29.6%)	0	(0%)	
Postpartum	NA		NA		NA		16	(59.3%)	0	(0%)	
Gestational period at the onset^e^											
Before labor, gestational age (day)	267	[255–281]	NA		NA		231	[203–288]	242	[191–271]	< 0.05^f^
During labor, gestational age (day)	NA		277	[261–287]	NA		278	[179–292]	NA		0.63
Postpartum, time after delivery (min)	NA		NA		1086	[484–1697]	60	[0–240]	NA		< 0.0001
Clinical symptoms (case)											
Cardiac arrest	NA		NA		NA		9	(33.3%)	0	(0%)	< 0.05
Respiratory failure	NA		NA		NA		4	(14.8%)	0	(0%)	0.29
PPH	NA		NA		NA		23	(85.2%)	9	(75.0%)	0.65
NRFS	NA		NA		NA		14	(51.9%)	8	(66.7%)	0.49
IUFD	NA		NA		NA		2	(7.4%)	4	(33.3%)	0.06
Time of the onset to blood collection (min)	NA		NA		NA		60	[0–200]	105	[0–360]	0.21
Blood loss at blood collection (mL)	0	[0–0]	0	[0–0]	350	[180–965]	4900	[880–7000]	2648	[430–5140]	< 0.0001^g^
Fluid amount at blood collection (mL)	0	[0–0]	0	[0–0]	600	[600–1300]	1300	[0–5500]	1355	[0–6000]	< 0.0001^h^
Maternal death (case)	NA		NA		NA		4	(14.8%)	0	(0%)	0.29
Neonatal death (case)	NA		NA		NA		2	(7.4%)	5	(41.7%)	< 0.05

Continuous data are shown as medians with minimum to maximum. Categorical data are represented as numbers with percentages. ^a^Post hoc multiple comparisons showed a significant difference between Postpartum in the control and AFE groups (P < 0.01). ^b^Post hoc multiple comparisons showed significant differences between During labor in the control and sPA groups (P < 0.05), Postpartum in the control and sPA groups (P < 0.05), as well as the AFE and sPA groups (P < 0.01). ^c^Residual analysis showed that During labor in the control group had significantly more vaginal deliveries (P < 0.01) and that the sPA group had significantly more Cesarean deliveries (P < 0.01). ^d^Residual analysis showed that the sPA group had significantly more cases of Before labor (P < 0.01) and that the AFE group had significantly more patients of During labor (P < 0.05) and Postpartum (P < 0.01). ^e^The gestational age or time after delivery at blood collection was described in the control group. ^f^Post hoc multiple comparisons showed a significant difference between Before labor in the control and sPA groups (P < 0.05). ^g^Post hoc multiple comparisons showed significant differences between Before labor in the control and AFE groups (P < 0.0001), Before labor in the control and sPA groups (P < 0.01), During labor in the control and AFE groups (P < 0.0001), During labor in the control and sPA groups (P < 0.01), as well as Postpartum in the control and the AFE groups (P < 0.05). ^h^Post hoc multiple comparisons showed significant differences between Before labor in the control and AFE groups (P < 0.001), Before labor in the control and sPA groups (P < 0.001), During labor in the control and AFE groups (P < 0.01), as well as During labor in the control and sPA groups (P < 0.01). *AFE* amniotic fluid embolism, *sPA* severe placental abruption, *NA* not applicable, *min* minute, *PPH* postpartum hemorrhage, *NRFS* non-reassuring fetal status, *IUFD* intrauterine fetal death.

**Table 2:** Hematological parameters.

	Control							AFE (n = 27)		sPA (n = 12)		P value
	Before labor (n = 9)			During labor (n = 7)		Postpartum (n = 7)						
Hemoglobin (g/L)	104		[84–119]	106	[87–139]	103	[95–117]	92	[10–136]	86	[43–126]	< 0.05^a^
Platelet count (× 10^9^/L)	254		[173–346]	245	[148–409]	198	[183–325]	120	[28–258]	117	[53–158]	< 0.0001^b^
PT-INR	0.89		[0.83–0.98]	0.90	[0.84–1.01]	0.92	[0.81–0.97]	2.02	[0.94–7.26]	1.15	[0.90–2.14]	< 0.0001^b^
Fibrinogen (g/L)	4.08		[3.42–4.51]	4.58	[3.82–5.49	3.87	[3.30–4.66]	0.50	[0.20–1.59]	0.90	[0.22–2.90]	< 0.0001^b^
D-dimer (µg/mL)	1.9		[1.4–3.1]	3.5	[2.2–4.2]	5.9	[2.7–12.1]	300.0	[4.0–1484.0]	107.9	[9.7–490.0]	< 0.0001^b^
Overt DIC defined by Erez^c^ (case)	NA			NA		NA		27	(100%)	12	(100%)	> 0.99
Overt DIC defined by Clark^d^ (case)	NA			NA		NA		20	(74.1%)	5	(14.7%)	0.07

Continuous data are shown as medians with minimum to maximum. Categorical data are represented as numbers with percentages. ^a^Kruskal–Wallis test, a non-parametric analysis, among whole five groups showed a significant difference, however, post hoc multiple comparison test did not show significant difference between each two groups. ^b^Post hoc multiple comparisons showed no significant difference between the AFE and the sPA group. ^c^Overt DIC defined by Erez was evaluated according to a previous study^30^. ^d^Overt DIC defined by Clark was evaluated according to a previous study^47^. *AFE* amniotic fluid embolism, *sPA* severe placental abruption, *PT-INR* prothrombin time-international normalized ratio, *DIC* disseminated intravascular coagulation.

### Coagulation and fibrinolysis factors of interest and their correlations

**Figure 1:**
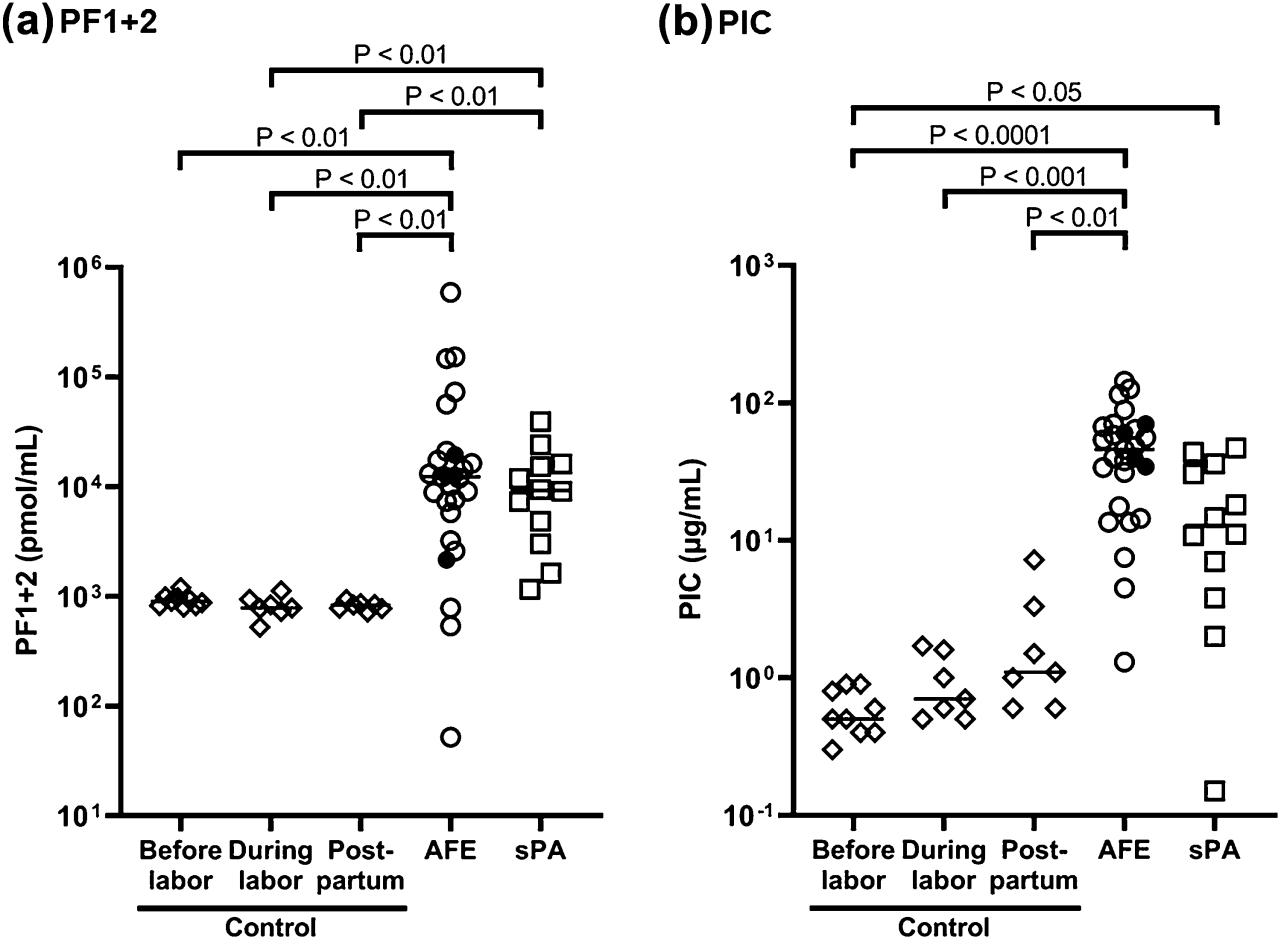
(**a**) Prothrombin fragment 1 + 2 (PF1 + 2) and (**b**) Plasmin α2-plasmin inhibitor complex (PIC) among groups of control, amniotic fluid embolism (AFE), and severe placental abruption (sPA). Both PF1 + 2 and PIC levels in AFE and sPA groups were significantly increased compared to the control; however, there were no significant differences between the AFE and sPA groups. Filled circles show fatal cases in the AFE group in this and all the following figures.

**Figure 2:**
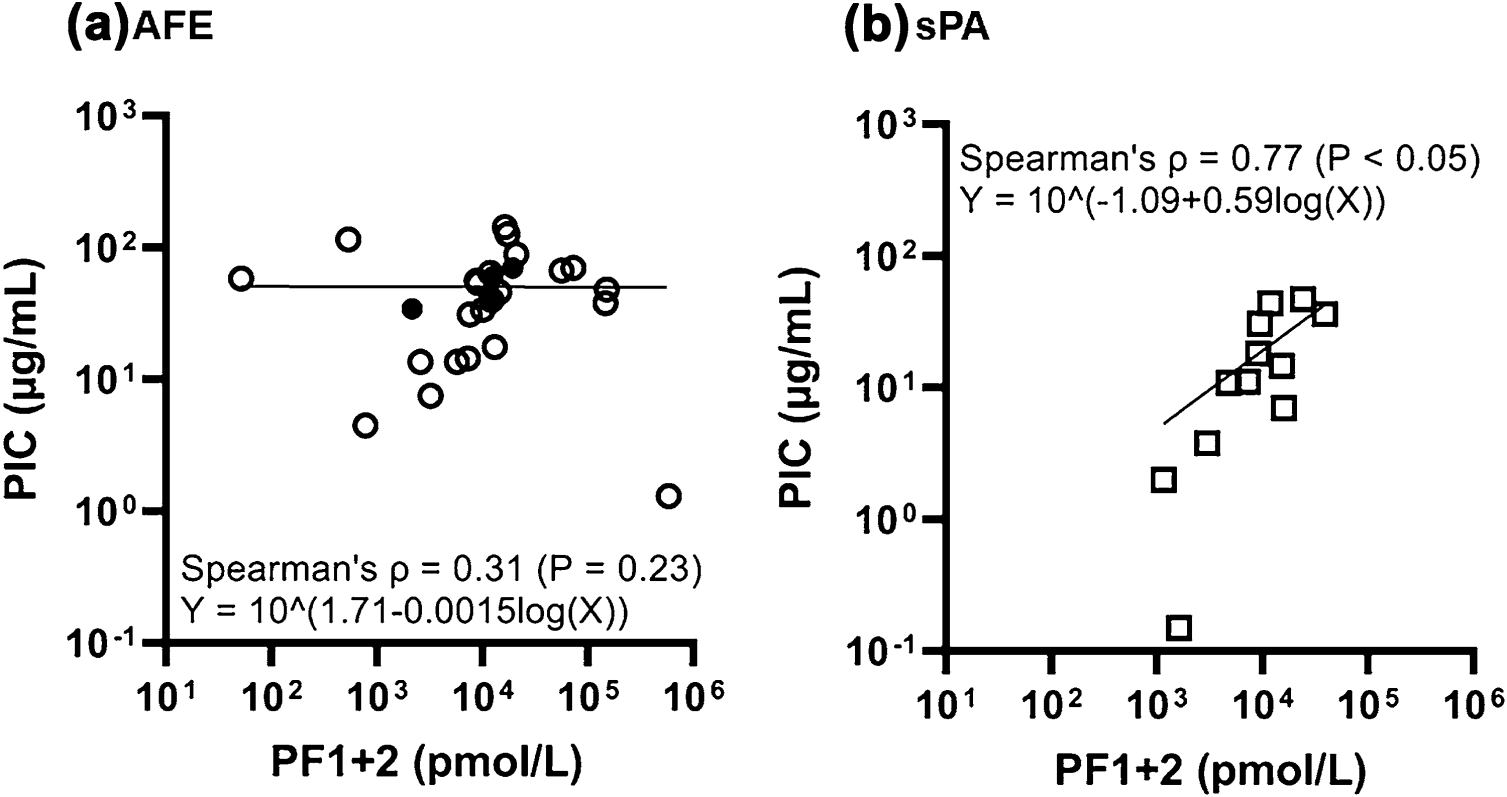
Correlation between PF1 + 2 and PIC in the (**a**) AFE and (**b**) sPA groups. There was no significant correlation between PF1 + 2 and PIC in the AFE group, whereas a positive correlation was found in the sPA group. We found a larger increase in plasma levels of PIC compared to those of PF1 + 2 in AFE patients in comparison with sPA patients.

**Figure 3:**
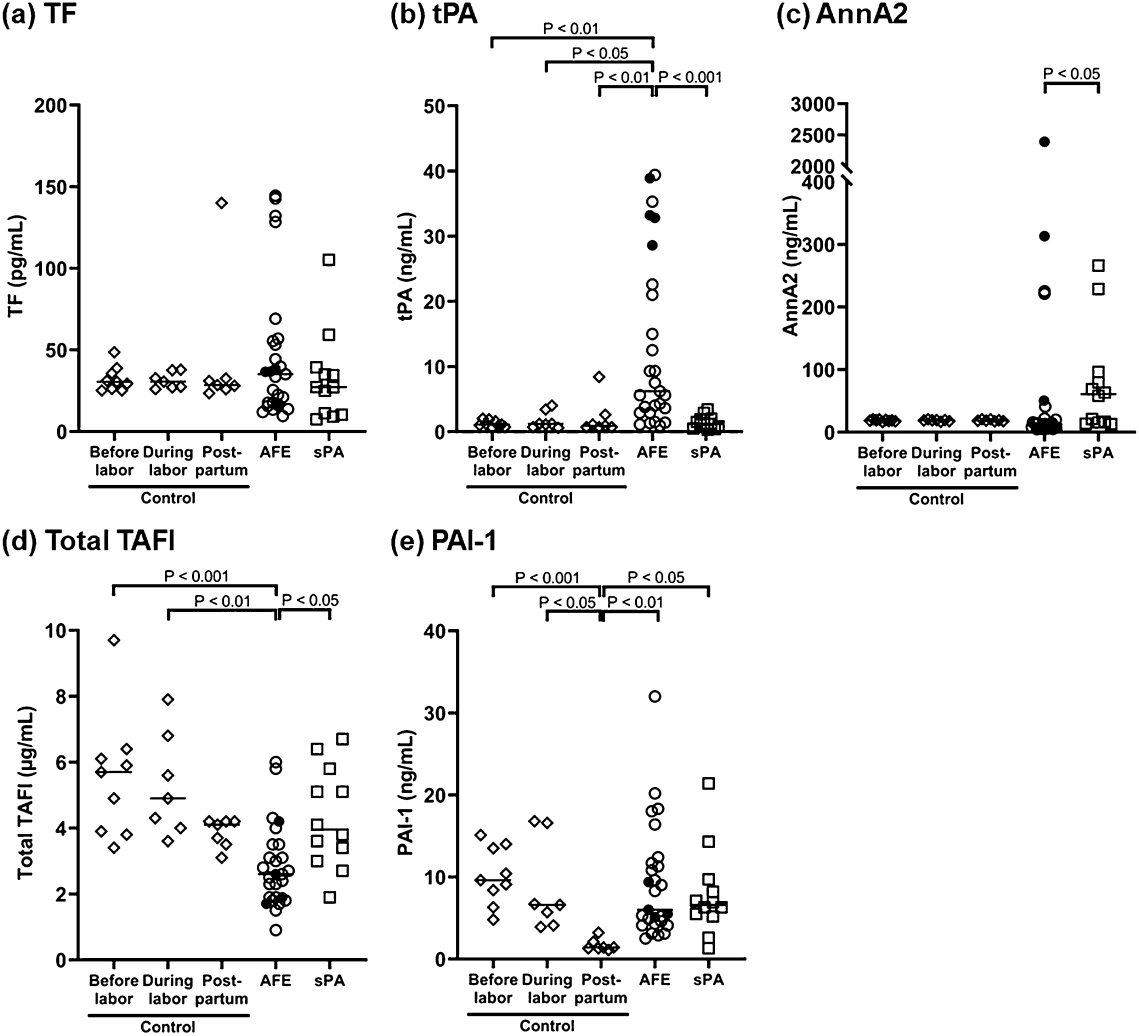
Coagulation and fibrinolysis factors of interest in the control, AFE, and sPA groups. We evaluated (**a**) tissue factor (TF), (**b**) tissue plasminogen activator (tPA), (**c**) annexin A2 (AnnA2), (**d**) total thrombin activatable fibrinolysis inhibitor (total TAFI, which included TAFI and TAFIa), and (**e**) plasminogen activator inhibitor-type 1 (PAI-1). The AFE group had significantly increased plasma levels of tPA and decreased total TAFI. AnnA2 levels were significantly higher in sPA than AFE patients; however, there were two fatal cases with the high levels of AnnA2 in the AFE group.

**Figure 4:**
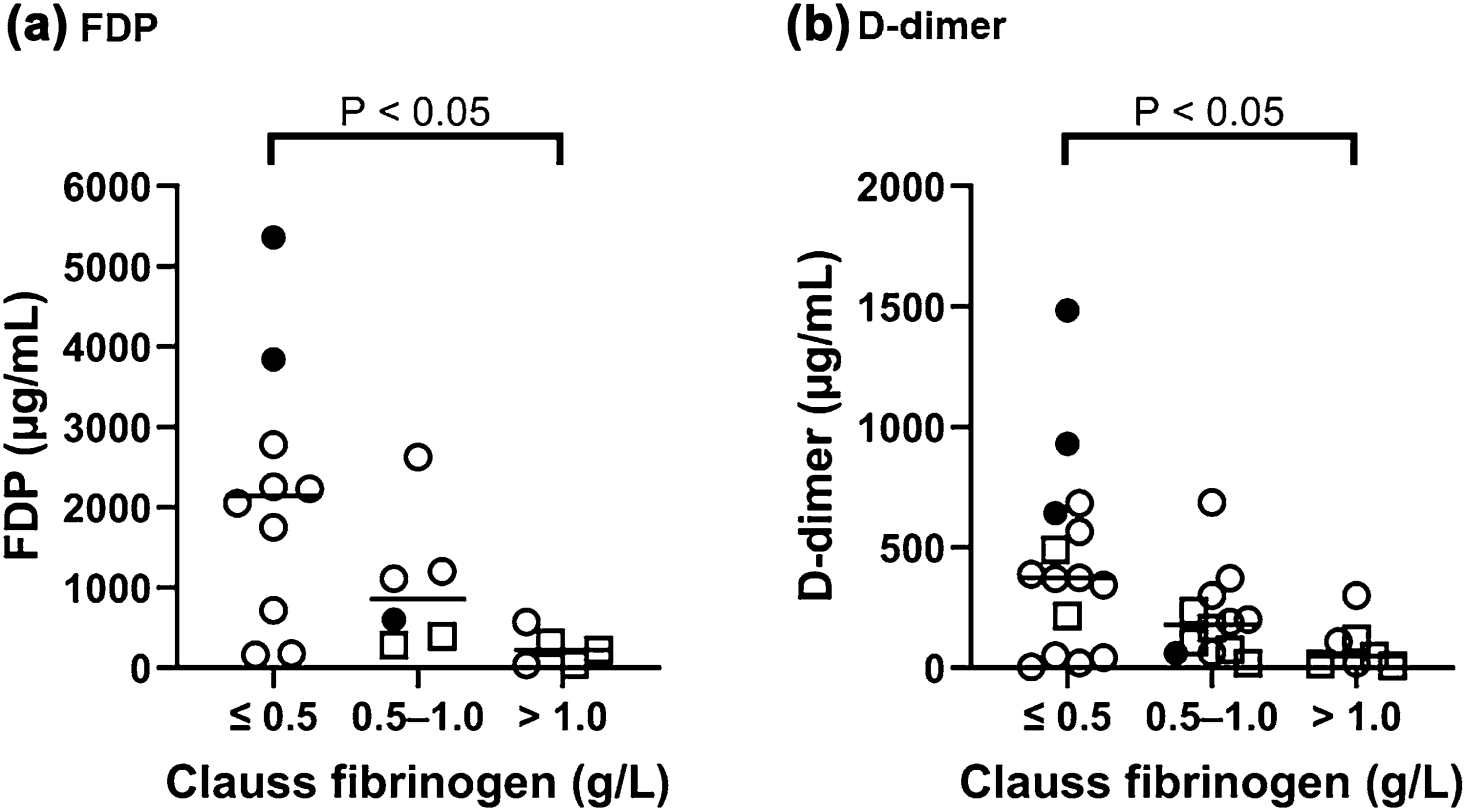
(**a**) Fibrin degradation products (FDP) and (**b**) D-dimer classified with Clauss fibrinogen levels in AFE and sPA groups. White circles and squares represent the AFE and sPA patients, respectively. Filled circles show fatal cases in the AFE group.

PF1 + 2 and PIC levels were higher in the AFE and sPA groups compared to the control group; however, there was no significant difference between the AFE and sPA groups (Fig. 1). Figure 2 shows correlations between PF1 + 2 and PIC in the AFE and sPA groups. PF1 + 2 was significantly correlated with PIC in sPA patients (ρ = 0.77, P < 0.05), but not in AFE patients (ρ = 0.31, P = 0.23). The plasma levels of PIC were increased compared to those of PF1 + 2 in AFE patients (Fig. 2a), in comparison with the distribution of these values and the regression curve in sPA patients (Fig. 2b). Notably, two out of three AFE cases that had less than 1000 pmol/L of PF1 + 2 levels, the same level as the control group, increased their PIC levels to around 100 µg/mL. Figure 3 shows the coagulation and fibrinolysis factors of interest among the five groups including three peripartum control subgroups. The AFE group had significantly increased levels of tPA (Fig. 3b) and decreased total TAFI (Fig. 3d) compared to the control and sPA groups. The AnnA2 level in the sPA group was significantly higher than that in the AFE group; however, there were two fatal cases with the high levels of AnnA2 in the AFE group (Fig. 3c). PAI-1 levels in Postpartum of the control group were significantly lower than the other subgroups in the control, AFE, and sPA groups (Fig. 3e). We did not find any significant difference in TF levels among the control, AFE, and sPA groups (Fig. 3a). The AFE and sPA subjects with very low values of Clauss fibrinogen (< 0.5 g/L) had significantly higher fibrin degradation products (FDP) and D-dimer levels than those with Clauss fibrinogen levels more than 1.0 g/L (Fig. 4).

## Discussion

Both AFE and sPA were associated with activation of coagulation and massive fibrinolysis without significant difference in each plasma maker between the groups. However, we found that fibrinolysis in AFE showed hyperactivation without correlating to the enhanced coagulation, compared to the significant positive correlation between activated coagulation and hyperfibrinolysis in sPA. The contribution of a significant increase of tPA on producing plasmin in plasma, as well as decrease of TAFI and/or TAFIa may combine to promote fibrinolytic hyperactivity, which is associated with the specific activation pathway of the fibrinolytic potential in AFE.

AFE and PA are potentially fatal obstetric diseases. They may be complicated with DIC. It has been reported that DIC has a common pathophysiology of persistent widespread activation of coagulation; however, the degree of fibrinolytic activation varies depending on underlying diseases^13^. Based on this fundamental concept and the reported different tracings of fibrinolytic phases in TEG or ROTEM between AFE^6,7^ and PA^9,10^ complicated with DIC, we focused on the specific blood coagulation and fibrinolysis cascade that could induce hyperfibrinolysis in AFE compared to sPA. We found that PF1 + 2 and PIC, the main markers employed in the study for evaluation of activated coagulation and fibrinolysis, respectively, were markedly increased in both the AFE and sPA groups (Fig. 1). The plasma levels of these groups were higher than the reported values in acute traumatic coagulopathy^14,15^. The D-dimer levels, another marker for fibrinolysis, also elevated significantly and the Clauss fibrinogen levels markedly decreased in those two groups (Table 2). Although there was no statistically significant difference in the blood loss between the AFE (4.9 L) and the sPA group (2.6 L) at the timepoint of blood samples drawn, there was a major clinically significant difference, which would impact on coagulation findings such as the difference in PT-INR between the AFE and sPA group. These data showed that both AFE and sPA were indeed associated with hyperactivation of blood coagulation and fibrinolysis without significant difference in each plasma maker between the groups, resulting in clinically significant hemostatic failure; however, AFE specifically induced the elevation of fibrinolytic potential which was not correlated with enhanced blood coagulation. On the other hand, fibrinolysis activation in sPA was positively correlated with coagulation activation (Fig. 2). We evaluated antigen levels, not activity, for the plasma tPA and total TAFI, therefore, not all of the high tPA and low total TAFI levels detected in our study contributed to plasmin production. However, the significantly increased tPA in AFE patients may contribute, at least partly, to enhanced fibrinolytic potential, whereas the inhibitory system in fibrinolysis could be impaired partly due to decreased level of total TAFI. As TAFI and TAFIa are also inhibitors of complement C3a and C5a^16^, the decrease of total TAFI may be associated with consumption to suppress the activated complement system, which we previously reported as the underlying pathophysiology in AFE^2,17,18^. These changes in fibrinolytic system could be combined to promote a hyperfibrinolytic state, which would underlie enhanced fibrinolytic activity that was not correlated with coagulation activation in AFE, in contrast with sPA. The plasma AnnA2 levels were higher in the sPA group than those in the AFE group, while we found two fatal AFE cases with the high AnnA2 levels. AnnA2 has been reported to be expressed in placental amniotic cells, vascular endothelial cells, chorionic villi, extravillous trophoblast, and decidua cells^19^ and to be up-regulated in response to hypoxic stimuli in retina of an animal model^20^. The significant increase of AnnA2 plasma levels might be associated with cell injury and/or stimulation as well as tissue structure disruption due to retroplacental hematoma in the sPA group, and also with hypoxic stimuli specifically in fatal AFE cases, resulting in a release of AnnA2 into maternal circulation. The contribution of AnnA2 to fibrinolysis in the sPA group remains to be clarified, however, it does not at least have sufficient fibrinolytic potential to produce a large amount of plasmin that would disrupt the positive significant correlation between PF1 + 2 and PIC shown in the sPA group (Fig. 2).

The plasma antigen levels of TF were not significantly different among peripartum control, AFE, and sPA groups. The activity-based assay for TF^21^ and evaluation of TF pathway inhibitor (TFPI), an inhibitory factor of activated TF pathway that was compromised in DIC^22^, might be more sensitive to find any differences among the groups. We speculated that the other factors may also have a possible contribution to coagulation activation such as amniotic fluid including activated factor X^23^ and other coagulation factors participating in the contact activation pathway^24^. Our previous in vitro study reported that amniotic fluid accelerated blood coagulation without inducing hyperfibrinolysis^25^. Furthermore, as the specific serine protease in the upstream of the complement system was reported to have factor X-like potential that can produce thrombin leading to generation of a fibrin clot^26^, an activated complement system, which was associated with the underlying pathophysiology in AFE^2,17,18^, may also enhance blood coagulation by the activated protease as another pathway. We did not find significant difference in PAI-1 levels between the AFE and sPA group but only a physiological decrease in Postpartum subgroup of the peripartum control^27,28^. The plasma level of PAI-2, i.e., another PAI produced in the placenta, has been reported to increase as the pregnancy advanced, reaching a peak at delivery^27,28^. It is also reported that amniotic fluid obtained during delivery contains abundant urokinase-type PA (uPA)^29^. During parturition, uPA is supposed to flow into maternal circulation through exposure to amniotic fluid. Evaluation of the plasma levels of these fibrinolytic and anti-fibrinolytic factors that have pregnancy-specific sources may provide further findings to clarify the similarity and difference in the fibrinolytic cascade between AFE and sPA, nonetheless, these factor levels are not available in the present study since we did not design to measure the factors due to the limited amount of plasma available.

In the present study, we used the modified-International Society on Thrombosis and Haemostasis score proposed by Erez^30^ to diagnose DIC in our AFE and PA cohorts; thereby we showed that AFE and sPA complicated with DIC had marked activation of coagulation and fibrinolysis and that AFE patients were affected by hyperfibrinolysis without correlating to the enhanced coagulation, which might be associated with the increased and decreased plasma levels of tPA and total TAFI, respectively. On the other hand, de Lloyd et al. reported that a small number of pregnant women (1.06/1000 maternities as the frequency) was associated with extremely increased PIC levels more than 40 µg/mL (hyperfibrinolysis) and decreased Clauss fibrinogen/fibrinogen antigen ratio (dysfibrinogenemia) due to AFE, PA, and postpartum hemorrhage caused by uterine atony, surgery/genital tract trauma, and placenta accreta/previa, and proposed the pathophysiology as acute obstetric coagulopathy^31^. They speculated that significantly increased FDP and D-dimer interfered polymerization of fibrin in this coagulopathy, resulting in a discrepancy between the functional and antigenic fibrinogen level, as described also in AFE case report^32^. We could not calculate the Clauss fibrinogen/antigenic fibrinogen ratio because our study was not designed to evaluate fibrinogen antigen levels when we started our study. However, the AFE and sPA patients included in the present study also showed very low Clauss fibrinogen levels with a significant increase in FDP and D-dimer values (Fig. 4). According to two previous in vitro studies^33,34^, we speculated that both fibrinogenolysis caused by the high level of plasmin and inhibition of fibrin polymerization by FDP might be associated with the relationship that the patients with the low Clauss fibrinogen had significantly high levels of FDP and D-dimer. Thus, coagulopathy in AFE and sPA is supposed to include a variety of pathophysiology involving activation of coagulation and fibrinolysis; therefore, further accumulation and analysis of cases may be needed to clarify the mechanism of the coagulopathy and ameliorate the diagnostic criteria for more accurate diagnosis of the focused population.

Antifibrinolytic treatment is considered to be critically important in the earlier stages of management of DIC in AFE to suppress hyperfibrinolysis. Tranexamic acid, a universally available antifibrinolytic agent, inhibits plasminogen from binding with fibrin and suppresses production of plasmin^35^. It has been reported to reduce death due to bleeding with postpartum hemorrhage^36^ and severe trauma^37^; however, its survival benefit sustains for only three hours from the onset of bleeding^38^. Cryoprecipitate is a blood-derived product, which is produced by thawing fresh frozen plasma (FFP) at 1–6 °C and removing the supernatant after centrifugation^39^. It contains α2-plasmin inhibitor^39,40^, one of the major antifibrinolytic factors. A study reported that transfusion of cryoprecipitate increased plasma PAI-1 levels and restored fibrin structure with increasing fiber toughness and stiffness in severe trauma patients with hemorrhage^41^. Earlier identification of hypofibrinogenemia is the most clinically important and correcting the low fibrinogen levels would be the highest priority in any management strategy. Fibrinogen concentrate can increase plasma fibrinogen value efficiently avoiding further risks of volume overload and pulmonary edema^42^; however, it lacks antifibrinolytic effect. Therefore, administration of fibrinogen concentrate for coagulation factors replacement should be combined with intravenous infusion of tranexamic acid and/or transfusion of blood products, such as FFP and cryoprecipitate, in management of hyperfibrinolytic DIC involving AFE.

There were several limitations in the present study. Some hematological values in the patients’ clinical information were above or below thresholds; therefore, we may have underestimated the severity of the coagulation disturbance. Since we did not design our study to measure the levels of the following factors due to the limited amount of each blood sample, the values of fibrinogen antigen, TFPI, uPA, and PAI-2, which might provide further significant findings on the mechanism of activated coagulation and fibrinolysis, could not be evaluated in the study. We could not reexamine samples after dilution with extremely high or low PF1 + 2 and PIC levels due to the small amount of blood samples available. Although the ELISA kits used in this study and the assays performed in the contract laboratory company were reliable, there might be some samples that could not be detected correctly for the PF1 + 2 and PIC levels due to an issue with the sample itself, which might affect the result of correlation analysis as outliers. We employed the antigen-based assay for evaluation of the TF level in plasma, which might be less sensitive than activity-based assays^21^. We evaluated tPA and TAFI by antigenic values, which might not accurately reflect their activity. As the peripartum control group was divided into three subgroups, the number of control subjects in one subgroup was small. Although we reviewed the clinical information of a total of 2059 cases from the Japan AFE Registry database between August 2009 and December 2021, the number of patients with citrated plasma specimens before blood transfusion available for the study was only 27 in the AFE group, and only 12 sPA patients with DIC. The cases in sPA group were highly selected population complicated with distinct coagulopathy; therefore, we could not extrapolate our findings and speculation to the whole PA patients. Although the number of the patients was small, it is notably important that these citrated plasma samples are significant, considering that the diseases covered in the present study are rare and that our department is the only institution in the world which has accumulated the blood specimens from AFE patients for 20 years long as far as we know^43^.

In conclusion, both AFE and sPA were associated with hyperactivation of coagulation and fibrinolysis with no significant difference in the markers between the groups. However, AFE showed a tendency to have increased fibrinolytic activation relative to the enhanced coagulation compared to PA. An increased tPA level and impairment of fibrinolytic inhibitory system due to a decreased TAFI/TAFIa level might be associated with further plasmin production in AFE, which could underlie the unique activation pathway of the fibrinolytic potential in AFE compared to PA.

## Methods

### Diagnosis of AFE, PA, and overt DIC

AFE was diagnosed using the Japanese AFE diagnostic criteria^2^. The definition requires one of the following symptoms to develop during pregnancy or within 12 h of delivery and thereby need intensive care: (1) cardiac arrest, (2) severe bleeding of unknown origin within 2 h of delivery (≥ 1500 mL), (3) DIC, or (4) respiratory failure (Supplementary Table S2). In the present study, placental abruption was diagnosed clinically and pathologically with evidence of retroplacental hematoma. Its clinical features vary depending on the location of retroplacental hematoma and placental abruption area^44^; therefore, we included PA patients complicated with DIC, which were consistent with Grade III in the classification of Page et al.^3^.This highly selected PA population that met with the following DIC diagnostic criteria was defined as severe PA (sPA) group in the present study. For the diagnosis of DIC, we employed modified DIC scores for pregnant women as defined by Erez^30^ based on the International Society on Thrombosis and Haemostasis scoring system. The score was calculated using platelet count, prothrombin time difference in seconds (referring to prothrombin time-international normalized ratio [PT-INR]), and fibrinogen level. A total score of 26 or more was diagnosed as overt DIC (Supplementary Table S3). We employed the diagnostic criteria reported by the International Society for the Study of Hypertension in Pregnancy^45^ to diagnose preeclampsia as a complication.

### Patients and blood samples

After our department launched the Japanese AFE Registry Program in 2003, clinical information and blood samples obtained from suspected AFE cases have been delivered to our department for the further auxiliary analysis of AFE^2^. Physicians are recommended to draw the patient’s blood during the clinical course when they suspect AFE. The blood samples are centrifuged to produce serum and/or plasma in each hospital and delivered frozen (− 30  °C) to our department with the patient’s written informed consent.

In the present case–control study, we reviewed AFE patients who met Erez’s DIC diagnostic criteria^30^ (Supplementary Table S3) in the Japanese AFE Registry database between August 2009 and December 2021 and included patients with a sufficient amount of blood specimens before transfusion. We included sPA complicated with DIC that was diagnosed using Erez’s criteria^30^ (Supplementary Table S3) who were managed and treated in Hamamatsu University Hospital between January 2016 and June 2021 with blood samples before transfusion available. As a control, we recruited normal pregnant participants at the subgroups of three peripartum stages: before labor, during labor, and postpartum until 30 h from delivery among singleton pregnancies without hematological complications involving platelets, blood coagulation, or postpartum hemorrhage in Hamamatsu University Hospital between January 2021 and June 2021. We defined postpartum hemorrhage as estimated bleeding amount of more than 800 and 1500 mL in vaginal and cesarean deliveries, respectively, according to Japanese practice guidelines^46^. In our hospital, 4 mL of whole blood were collected into a vacutainer containing 3.2% sodium citrate during venipuncture at the time of the insertion of a 20 G intravenous catheter or a blood test clinically indicated in admission, delivery, and postpartum period. The whole blood samples were centrifuged at 3000 rounds per minute for 10 min and the supernatant plasma was preserved frozen at − 30  °C in aliquots for analysis.

### Data collection and measurement of coagulation and fibrinolysis factors of interest

We collected hematologic data of hemoglobin concentration, platelet counts, fibrinogen levels (Clauss method), PT-INR, FDP, and D-dimer in all groups. We measured FDP (Nanopia P-FDP, SEKISUI MEDICAL, Tokyo, Japan) and D-dimer (LATECLE D-dimer, KAINOS, Tokyo, Japan) as necessary. The FDP assay is sensitive to both fibrin and fibrinogen degradation products. Overt DIC was evaluated with the two scoring systems and the numbers of the DIC patients were calculated. All AFE and PA patients met with the DIC diagnostic criteria defined by Erez^30^ (Supplementary Table S3) along with our inclusion criteria; furthermore, the DIC diagnostic criteria for pregnancy reported by Clark^47^ (Supplementary Table S4) were used. A comparison of the diagnostic criteria defined by Erez and Clark is shown in Supplementary Table S5.

We also evaluated the following variables in plasma specimens among the three groups: prothrombin fragment 1 + 2 (PF1 + 2) (Enzygnost F1 + 2 monoclonal, Siemens Healthineers, Erlangen, Germany) and plasmin-α2 plasmin inhibitor complex (PIC) (LPIA-ACE PPI II, LSI Medience, Tokyo, Japan). PF1 + 2 is produced in blood when prothrombin is converted to thrombin by activated Factor X. PIC, also described as plasmin/antiplasmin complex (PAP) elsewhere, measures the amount of plasmin, which is a major fibrinolytic enzyme in blood. We measured the antigen amounts of the coagulation and fibrinolysis factors of interest using enzyme-linked immunosorbent assays (ELISA) as follows: tissue factor (TF) (ab108903, abcam, Cambridge, UK) as an initiator of blood coagulation by forming a complex with activated Factor VII; tissue plasminogen activator (tPA) (ab108914, abcam, Cambridge, UK), which can activate plasminogen efficiently on the surface of fibrin clot; annexin A2 (AnnA2) (SEB944Hu, Cloud Clone, Houston, TX, USA), which bridges tPA and plasminogen as a receptor, leading to activation of plasminogen thereby producing plasmin; plasminogen activator inhibitor-type 1 (PAI-1) (Human Serpin E1/PAI-1, R&D systems, Minneapolis, MN, USA), which forms a complex with tPA and inhibits plasminogen activation in plasma; and thrombin activatable fibrinolysis inhibitor (TAFI) (ab272774, abcam, Cambridge, UK), a fibrinolytic inhibitor that is activated efficiently by thrombin–thrombomodulin complex and converted to its activated form (TAFIa) by thrombin and plasmin^16^. TAFIa cleaves arginine and lysin residues in the C-terminus of the fibrin and inhibits plasminogen and plasmin binding to the fibrin clot. Since the capture antibody in the ELISA kit used in the present study detects both TAFI and TAFIa antigens according to the manufacturer, we described the amount of TAFI and TAFIa measured together with the kit as total TAFI. The assays of D-dimer and PIC were performed in SRL Inc., Tokyo, Japan. According to the previous report from the Scientific and Standardization Committee of the International Society on Thrombosis and Haemostasis^21^, activity-based assays are more sensitive and may be more reliable than antigen-based assays for measuring TF in plasma samples, however, we employed an antigen-based assay for evaluation of the TF level because our research project did not include the evaluation of TF activity when we started our study before the report available.

### Statistical analysis

When the patients’ data were above or below the threshold of the assay and could not be identified as exact values, we described each threshold instead. All continuous data were described as medians with a minimum to maximum. We employed Fisher’s exact test or chi-squared test for categorical data. Continuous data were evaluated by non-parametric analyses such as Mann–Whitney *U* test between two groups and a Kruskal–Wallis analysis followed by Dunn’s multiple comparison test among five groups including three peripartum control subgroups. We calculated Spearman’s correlation coefficient, described as ρ (rho), to examine the relationship between parameters. Nonlinear regression curve was depicted in each graph with the regression equation. A two-sided P value < 0.05 was defined as significant. P values were adjusted to account for multiple comparisons. GraphPad Prism version 9 (GraphPad Software Inc., CA) was used for statistical calculations. The Institutional Review Board at the Hamamatsu University School of Medicine approved the present study (No. 15-333 and 16-165). All researches were performed in accordance with relevant guidelines and regulations which was designated by the Institutional Review Board at the Hamamatsu University School of Medicine. We obtained written informed consent from all patients.

### Supplementary Information

Supplementary Tables.

## Data Availability

The data that support the findings of this study are available from the corresponding author, T.O., upon reasonable request.
