# Supplementary material for: Comparative analysis of hyperfibrinolysis with activated coagulation between amniotic fluid embolism and severe placental abruption
Source: Sci Rep. 2024 Jan 2;14:272. doi: 10.1038/s41598-023-50911-w (PMC10761968; doi:10.1038/s41598-023-50911-w)
Supplement: Supplementary file 1 — Supplementary Tables. [file 41598_2023_50911_MOESM1_ESM.pdf]

**Table S1.** The number of cases complicated with preeclampsia.

| **Group** | **AFE** | | **sPA** | |
|---|---|---|---|---|
| Enrolled in the Registry | 2,059 | | NA | |
| Preeclampsia | 99 | (4.8%) | NA | |
| | | | | |
| DIC diagnosed by Erez's criteria | 789 | | 42 | |
| Preeclampsia | 28 | (3.6%) | 5 | (11.9%) |
| | | | | |
| Eligible cases for the study | 27 | | 12 | |
| Preeclampsia | 0 | (0%) | 1 | (8.3%) |

Preeclampsia was diagnosed with the diagnostic criteria reported by the International Society for the Study of Hypertension in Pregnancy[45].

Abbreviations: AFE, amniotic fluid embolism; sPA, severe placental abruption; DIC, disseminated intravascular coagulation, NA, not applicable.

**Table S2. Japanese diagnostic criteria for amniotic fluid embolism[2].**

| |
|---|
| (1) If symptoms appeared during pregnancy or within 12 h of delivery. |
| (2) If any intensive medical intervention was conducted to treat one or more of the following symptoms/diseases: |
| A) Cardiac arrest |
| B) Severe bleeding of unknown origin within 2 hours of delivery (≥ 1500 mL) |
| C) Disseminated intravascular coagulation |
| D) Respiratory failure |
| (3) If the findings or symptoms obtained cannot be explained by other diseases. |
| A clinical diagnosis of AFE can be made if the pathological condition meets the above three criteria. |
| Because these diagnostic criteria serve the purpose of making a clinical diagnosis and being able to promptly provide treatment, the pathological conditions that meet them may include those other than AFE. |

**Table S3. The diagnostic criteria of DIC in pregnancy proposed by Erez[30].**

| Parameters | Value | Score |
|---|---|---|
| PT difference (sec) | < 0.5 | 0 |
| | 0.5–1 | 5 |
| | 1.0–1.5 | 12 |
| | > 1.5 | 25 |
| Platelet count ($10^9$/L) | < 50 | 1 |
| | 50–100 | 2 |
| | 100–185 | 1 |
| | > 185 | 0 |
| Fibrinogen (g/L) | < 3.0 | 25 |
| | 3.0–4.0 | 6 |
| | 4.0–4.5 | 1 |
| | > 4.5 | 0 |
| Calculated score | | ≥ 26: diagnosed as DIC |

PT difference was calculated as the difference between the result of the patient and that of the laboratory normal control[30]. When the patient had only the data set of PT in seconds and PT-INR, we calculated the laboratory normal control value as the patient's PT in seconds divided by PT-INR. Abbreviations: DIC, disseminated intravascular coagulation; PT, prothrombin time; PT-INR, prothrombin time-international normalized ratio.

**Table S4. Diagnostic criteria of DIC in pregnancy proposed by Clark[47].**

| Parameters | Value | Score |
|---|---|---|
| Platelet count (/μL) | > 100,000 | 0 |
| | 50,000–100,000 | 1 |
| | < 50,000 | 2 |
| Prolonged PT or PT-INR | < 25% increase | 0 |
| | 25–50% increase | 1 |
| | > 50% increase | 2 |
| Fibrinogen level (mg/dL) | > 200 | 0 |
| | < 200 | 1 |
| Calculated score | | ≥ 3: compatible with overt DIC |

Abbreviations: DIC, disseminated intravascular coagulation; PT, prothrombin time; PT-INR, prothrombin time-international normalized ratio.

**Table S5. Comparison of two different DIC diagnostic criteria in pregnancy.**

| Parameters | Reported by Erez[30] | Score | Reported by Clark[47] | Score |
|---|---|---|---|---|
| Platelet count ($\times 10^9$/L) | > 185 | 0 | > 100 | 0 |
| | 100–185 | 1 | 50–100 | 1 |
| | 50–100 | 2 | < 50 | 2 |
| | < 50 | 1 | | |
| PT | PT difference (sec) | | Prolonged PT (sec) or PT-INR | |
| | < 0.5 | 0 | < 25% increase | 0 |
| | 0.5–1 | 5 | 25–50% increase | 1 |
| | 1.0–1.5 | 12 | > 50% increase | 2 |
| | > 1.5 | 25 | | |
| Fibrinogen (g/L) | > 4.5 | 0 | > 2.0 | 0 |
| | 4.0–4.5 | 1 | < 2.0 | 1 |
| | 3.0–4.0 | 6 | | |
| | < 3.0 | 25 | | |
| Diagnosis of DIC | ≥ 26 scores | | ≥ 3 scores | |

Abbreviations: DIC, disseminated intravascular coagulation; PT, prothrombin time; PT-INR, prothrombin time-international normalized ratio.
